# Following the ontogeny of retinal waves: pan-retinal recordings of population dynamics in the neonatal mouse

**DOI:** 10.1113/jphysiol.2013.262840

**Published:** 2014-01-31

**Authors:** Alessandro Maccione, Matthias H Hennig, Mauro Gandolfo, Oliver Muthmann, James van Coppenhagen, Stephen J Eglen, Luca Berdondini, Evelyne Sernagor

**Affiliations:** 1 Department of Neuroscience and Brain Technologies, Istituto Italiano di TecnologiaGenoa, Italy; 2 Institute for Adaptive and Neural Computation, School of InformaticsEdinburgh, UK; 3 Institute of Neuroscience, Newcastle University Medical SchoolNewcastle upon Tyne, UK; 4 Cambridge Computational Biology Institute, University of CambridgeCambridge, UK

## Abstract

The immature retina generates spontaneous waves of spiking activity that sweep across the ganglion cell layer during a limited period of development before the onset of visual experience. The spatiotemporal patterns encoded in the waves are believed to be instructive for the wiring of functional connections throughout the visual system. However, the ontogeny of retinal waves is still poorly documented as a result of the relatively low resolution of conventional recording techniques. Here, we characterize the spatiotemporal features of mouse retinal waves from birth until eye opening in unprecedented detail using a large-scale, dense, 4096-channel multielectrode array that allowed us to record from the entire neonatal retina at near cellular resolution. We found that early cholinergic waves propagate with random trajectories over large areas with low ganglion cell recruitment. They become slower, smaller and denser when GABA_A_ signalling matures, as occurs beyond postnatal day (P) 7. Glutamatergic influences dominate from P10, coinciding with profound changes in activity dynamics. At this time, waves cease to be random and begin to show repetitive trajectories confined to a few localized hotspots. These hotspots gradually tile the retina with time, and disappear after eye opening. Our observations demonstrate that retinal waves undergo major spatiotemporal changes during ontogeny. Our results support the hypotheses that cholinergic waves guide the refinement of retinal targets and that glutamatergic waves may also support the wiring of retinal receptive fields.

## Introduction

During perinatal development, neighbouring retinal ganglion cells (RGCs) fire in correlated bursts of spikes, resulting in propagating waves (Meister *et al*. 1991; for review see Torborg & Feller, 2005; Blankenship & Feller, 2010; Sernagor & Hennig, 2013). Although most studies have been performed *in vitro*, these findings have also been validated *in vivo* (Ackman *et al*. 2012; Siegel *et al*. 2012). The spatiotemporal features encoded in the waves are hypothesized to provide cues for the establishment of retinal receptive fields (Sernagor *et al*. 2001), as well as for eye-specific ON–OFF pathway segregation and visual map formation in retinal targets (Wong & Oakley, 1996; Wong, 1999; Lee *et al*. 2002; Akrouh & Kerschensteiner, 2013).

Although the hallmark of retinal waves is synchronized activity in neighbouring RGCs, this phenomenon is dynamic. In mammals, waves are produced in three consecutive stages with unique cellular and pharmacological signatures. They are first mediated by gap junctions (Stage I: late gestation) (Catsicas *et al*. 1998; Bansal *et al*. 2000; Syed *et al*. 2004a), and then depend on cholinergic synaptic transmission (Stage II: until P9–10 in mouse) (Feller *et al*. 1996; Sernagor & Grzywacz, 1996, 1999; Catsicas *et al*. 1998; Wong *et al*. 1998; Bansal *et al*. 2000; Sernagor *et al*. 2000; Zhou & Zhao, 2000; Sernagor *et al*. 2003; Syed *et al*. 2004b). GABAergic signalling becomes involved in modulating waves at around P4–5 in mouse (Zhang *et al*. 2006; Hennig *et al*. 2011) and at these early stages is depolarizing, as it is elsewhere in the developing central nervous system (CNS) (Ben-Ari *et al*. 2007), shifting polarity after P6 (Zhang *et al*. 2006; Barkis *et al*. 2010). Finally, glutamate signalling between bipolar cells takes over as the main driver of Stage III waves at around P10 in mouse (Bansal *et al*. 2000; Zhou & Zhao, 2000; Syed *et al*. 2004b; Blankenship *et al*. 2009), although gap junctions between ON bipolar cells are also involved in lateral transmission (Akrouh & Kerschensteiner, 2013).

Despite these fundamental developmental changes, there is currently no consensus on concomitant changes in activity dynamics. Stage II waves are characterized by slow propagation speeds, random initiation points and trajectories, and wide ranges in size and duration (Feller *et al*. 1997; Hennig *et al*. 2009, 2011), with a bias in the nasotemporal axis (Stafford *et al*. 2009). Precisely how GABA modulates Stage II waves is still largely unknown. In turtle, waves have been shown to become smaller and more stationary as GABA_A_ signalling matures (Sernagor *et al*. 2003). There are mixed reports on the properties of Stage III waves. They are rarer and either spatially restricted or stationary in rabbit (Syed *et al*. 2004b), but appear to have a frequency and speed similar to those of Stage II waves in mouse (Blankenship *et al*. 2009), although faster propagation has been reported (Demas *et al*. 2003). This incomplete characterization of the developmental changes in wave dynamics is likely to reflect the limited retinal coverage and spatial resolution of the multielectrode arrays (MEAs) and limited temporal resolution of the cellular imaging employed so far.

Here, we used the high-density, large-scale active pixel sensor (APS) MEA (Berdondini *et al*. 2009) to precisely examine how spatiotemporal properties of mouse retinal waves change on a day-by-day basis until eye opening. By recording simultaneously from 4096 electrodes covering an area of 7.13 mm^2^, we have achieved a novel, pan-retinal perspective on how early retinal spontaneous activity evolves as the retina matures.

## Methods

### Ethical approval

All experimental procedures were conducted in line with the UK Home Office Animals (Scientific Procedures) Act 1986. Initial experiments were performed in Genoa and conducted in accordance with the guidelines established by the European Community Council (Directive 2010/63/EU of 22 September 2010). Experimental protocols were approved by the Italian Ministry of Health.

### Tissue preparation and maintenance

Experiments were performed in neonatal (P2–15) C57bl/6 mice. Mouse pups were killed by cervical dislocation and eyeballs enucleated prior to retinal isolation. The isolated retina was placed, RGC layer facing down, onto the MEA (Fig. 1*A*). High-quality coupling between the tissue and electrodes was achieved by placing a small piece of polyester membrane filter (Sterlitech Corp., Kent, WA, USA) on the retina followed by a home-made anchor. The retina was kept in constant darkness at 32°C using an in-line heater (Warner Instruments LLC, Hamden, CT, USA) and continuously perfused using a peristaltic pump (∼1 ml min^−1^) with artificial cerebrospinal fluid (ACSF) containing 118 mm NaCl, 25 mm NaHCO_3_, 1 mm NaH_2_PO_4_, 3 mm KCl, 1 mm MgCl_2_, 2 mm CaCl_2_ and 10 mm glucose, equilibrated with 95% O_2_ and 5% CO_2_. Retinas were allowed to settle for 1–2 h before recordings were started in order to allow spontaneous activity to reach steady-state levels. Solutions were never recycled and thus the tissue was constantly perfused with fresh ACSF. Bicuculline metabromide (Tocris Bioscience, Bristol, UK) was added directly to the perfusate. In some experiments, retinas were kept alive for 2–3 days on the array. In these experiments, the in-line heater was turned off at the end of the first day of recordings and the retina was perfused overnight with fresh oxygenated ACSF at room temperature. On days 2–3, recordings were resumed about 1 h after the in-line heater had been turned on (Hennig *et al*. 2011).

**Figure 1:**
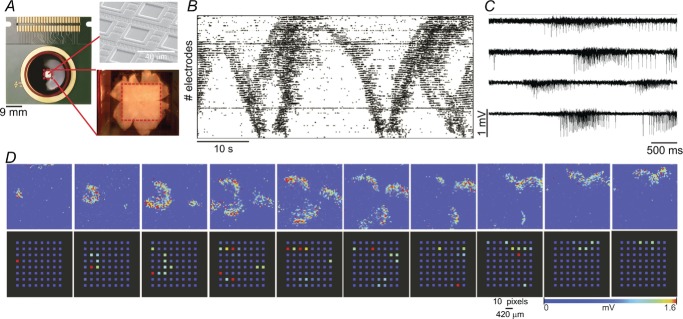
*A*, the APS MEA chip. The red dotted line demarcates the electrode area. Top inset, scanning electron micrograph illustrating the topography of individual electrodes on the chip. Bottom inset, magnification of the active area of the chip with a retina positioned on the electrodes. *B*, spike raster plot of spontaneous episode of activity in a P11 retina. *C*, raw signals on four sampled channels from the same recording. *D*, two-dimensional time-lapse (every 1 s for 10 s) view of the activity. The s.d. of the voltage is estimated in 10 ms bins and plotted using an exponential colour coding scheme to emphasize large deviations and effectively threshold small deviations. Bottom row: same episode after downsampling the resolution to a simulated 8 × 8 array with an electrode pitch of ∼334 *μ*m.

### Recording of retinal waves with the high-resolution electrode array platform

High-resolution extracellular recordings were performed on the BioCam4096 platform with APS MEA chips type BioChip 4096S (3Brain GmbH, Lanquart, Switzerland), providing 4096 square microelectrodes of 21 × 21 *μ*m in size (Fig. 1*A*) on an active area of 2.67 × 2.67 mm and with inter-electrode separation of 21 *μ*m. The platform records at a sampling rate of 7.8 kHz/electrode when measuring from the full 64 × 64 array (Imfeld *et al*. 2008). Raw data were visualized and recorded with the BrainWave software application provided with the BioCam4096 platform. Firing from the RGC layer was recorded at 12 bits resolution per channel, low-pass filtered at 8 kHz with the on-chip filter and high-pass filtered by setting the digital high-pass filter of the platform at 0.8 Hz. Each dataset generally consisted of 30 min of continuous recording at full-frame rate.

### Data analysis

The acquired raw data were first processed with the spike detection algorithm provided in the BrainWave software [precise timing spike detection (PTSD)] (Maccione *et al*. 2009) and by setting the s.d. at 7.5, the peak life time period at 2.0 ms, the refractory period at 1.0 ms, and enabling the option of noise level update during spike detection. Datasets of detected spike times were then exported for further analysis as Matlab files using the built-in export function in BrainWave. Files were then converted to HDF5 for further analysis in R. These HDF5 files are freely available for download on the CARMEN portal (https://portal.carmen.org.uk/). Instructions for accessing these files are available in the supporting information for this article (online: aps2014_hdf.pdf).

### Detection of retinal waves

Retinal waves were detected and analysed using custom algorithms written in Matlab (Version R2007A; MathWorks, Inc., Natick, MA, USA), based on a method originally developed for 60-channel MEA recordings (Hennig *et al*. 2009, 2011). The method consists of two steps: (i) bursts are detected on each electrode separately, and (ii) bursts are grouped into waves based on temporal overlap and proximity. Burst onsets were detected by ranking interspike intervals (ISIs) from the smallest to the largest values and selecting all intervals below a rank threshold of 0.75 as candidate bursts. To be considered as a burst, the spike count in the 1 s window following the first spike of the first interval below threshold rank had to exceed a threshold *θ*. The threshold *θ* was set to the spike count at the 95% mark of the cumulative spike count distribution, which was estimated from the histogram. This value was calculated individually for each channel to account for substantial variations in firing rate. The end of a burst was determined as the time when the spike count fell below *θ*/2. Only bursts with at least six spikes were included in the analysis. Waves were detected as temporally overlapping groups of bursts. To prevent splitting of waves during short bursts and merging of waves during long bursts, minimum and maximum burst durations of 2 s and 3 s, respectively, were imposed. To enable the distinction of multiple separate waves propagating across the retina simultaneously, a burst was included in a wave only if it was in spatial proximity to the previously included bursts. Specifically, for an active wave, the distances between the centres of electrodes that were recording successive bursts were calculated, and a new candidate burst was included in this wave only if this kept the variance of distances below a set threshold, which was manually adjusted for each recording. This method prevented active channels that belonged to spatially separate wave events from being included in a wave, and was robust against spatial inhomogeneities in the density of recorded channels. Trajectories were typically calculated over the past 30 burst events, and the threshold on the variance was adjusted manually to reflect the recorded cell density in each recording.

### Characterization of wave propagation

Detected waves were classified by means of estimating centre of activity trajectories (CATs) (Gandolfo *et al*. 2010). More specifically, a CAT was computed for each wave as the trajectory given by the time sequence of centre of activity (CA) points separated by a given time step (TS). Each CA point gives the coordinates of the geometric centre of the activity with respect to the electrode array, defined as the number of spikes identified in a given time window (TW). For retinal waves, we used TS = 1.0 s and TW = 1.5 s. To classify waves independently of their relative speed but only on the basis of their trajectories, CAT lengths were equalized by interpolating shorter trajectories with a spline function. The computed CATs were then classified by the *k*-means algorithm, which selected the best grouping among those obtained by varying *k* between 2 and 10 and by evaluating the silhouette width (Kaufman & Rousseeuw, 1990). Outliers identified as lying outside the Euclidean space centred in the cluster centroid and as having a radius of three times the s.d. of the cluster distribution were removed.

Wave speeds and lengths were computed using the original (non-interpolated) CATs. Wave length was computed as the Euclidian length of the trajectory (sum of the Euclidean distances between subsequent CA points). Wave speed was calculated as the ratio between the wave length and duration.

### Wave area and activity density

Wave area was determined by measuring the alpha convex hull around all electrodes that participated in a wave. The free parameter alpha determines the degree of convexity of the outline; here, this was set to 0.1 of the inter-electrode distance. The alpha convex hull was computed in the R programming environment using the alphahull package (Pateiro-López & Rodríguez-Casal, 2010). Activity density within a wave was defined as the number of active electrodes divided by the wave area.

### Correlation index

The correlation index (CI) (Wong *et al*. 1993) was used to calculate the correlation in spike firing between pairs of electrodes on the array, using a coincidence detection window of +/−50 ms. We considered only electrodes with bursting activity and an average firing rate in the range of 0.033–5.000 Hz as very high or low firing rates distorted the CI. The CI for a pair of electrodes was then plotted as a function of the distance separating the electrodes. The number of pairwise CIs rose quadratically with the number of electrodes and hence the plots of CI versus distance were visualized by binning the plots into equally sized hexagons (using the Hexbin package in R). We then computed the mean CI at a given range of distances (50 *μ*m bin width) and fitted a decaying exponential to these mean correlations:


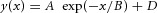


where *y*(*x*) is the mean CI at a given electrode separation *x* (usually measured out to 2 mm), and *A*, *B* and *D* are parameters that are found using non-linear least-squares estimation (routine nls within R). The baseline parameter *D* accounts for the expectation that uncorrelated spike trains should have a CI of 1. The parameter *A* is then interpreted as the ‘peak correlation’, and *B* is interpreted as the ‘decay length’, with larger values denoting correlations over longer distances. We then computed 95% confidence intervals for *A* and *B* using Monte-Carlo resampling (R = 100 simulations) of the CI values and recomputed the exponential fit for each simulation (Efron & Tibshirani, 1991).

### Activity hotspot detection: *P*-value

The aim of this analysis was to establish whether activity was evenly distributed over the retina or whether there were hotspots. To reliably detect active channels, we estimated the one-third and two-third quantiles of the raw voltage traces and isolated events that exceeded a set threshold (four times their difference below the one-third quantile). Persistent drops in voltage were ignored. To assess which channels show neuronal activity, we made use of the fact that the activity propagates in waves and estimated which channels were significantly correlated with the activity in their neighbourhood (*P* < 0.05, pooled data of all recordings).

As population activity was fairly constant over time, we ranked the spike times and performed all the analyses on spike ranks. For each channel, we calculated the number of occurrences of a spike in the 20 closest channels within a range of four spikes before or after each spike of that same channel. For random firing, the expected number of such coincidences between two channels is eight times the product of their spike counts divided by the population spike count, and follows a Poisson distribution.

The median over all 20 closest channels of *P*-values to accept that hypothesis of random firing was formed and visualized (hence the lowest *P*-values represent instances of highly correlated activity). As the statistics become generally better with a larger sample size (i.e. higher firing rates), we used this measure to visualize activity hotspots.

### Activity hotspots: firing similarity

As an alternative approach to visualizing the degree of correlation in the network, we calculated the rank differences of all spikes to the third closest spike on all other channels. After a normalization step to obtain a global average of 0.5 for the rank differences, we averaged them for each channel. The s.d. of these averages was used as a measure of how similarly a particular neurone fires with respect to the firing of the other neurones in the recording. We ranked these s.d. values in order to compare data from different recording sessions.

### Activity hotspots: correlation gradients

We used a method similar to that described for firing similarity, although this approach uses the variance within a radius of 0.2 mm of one channel (averaged over all channels as reference channels) and divides that value by the variance of neighbouring channels to find where correlations change and therefore where retinal waves tend to stop. This visualization method emphasizes areas of activity inhomogeneity, as well as boundaries between differentially active cells or clusters of cells (hotspots).

## Results

### Analysing high-density MEA recordings

The APS MEA allows simultaneous recording of neural activity from dissociated cultures and slices using 4096 closely spaced electrodes (Berdondini *et al*. 2009; Ferrea *et al*. 2012). The system uses 21 × 21 *μ*m electrodes with 42 *μ*m centre-to-centre separation, arranged in a 64 × 64 configuration (Fig. 1*A*). Each electrode consists of a small conductive recessed area surrounded by insulating walls (Fig. 1*A*, inset) contiguous with neighbouring electrodes. The array covers an active area of 2.67 × 2.67 mm, which is large enough to span the neonatal mouse retina, in which eye circumference measures ∼3 mm at P2 and ∼4 mm at P10 (Wisard *et al*. 2011). A global episode of spontaneous activity recorded in a P11 retina in the presence of the GABA_A_ antagonist bicuculline is illustrated in Fig. 1*B*–*D* (see also supporting information Movie S1, online). The raster plot, in which each row represents a different active channel and each dot an action potential, reveals complex propagation patterns (Fig. 1*B*), which are better seen in a two-dimensional time-lapse view of the activity (Fig. 1*D*, top row). These plots show the variations in voltage signal, in which the warmer colours indicate spiking (peak value: 1.6 mV) and intermediate colours indicate subthreshold responses. Good coupling is usually achieved between the tissue and the device, yielding large spikes with an excellent signal-to-noise ratio (Fig. 1*C*). We routinely record spikes that have amplitudes of hundreds of *μ*V, and it is not uncommon to see spikes with amplitudes of >1 mV, which is unusually large for extracellular MEA recordings. We attribute these large signals to the design of the electrodes, which causes the patch of retina within each electrode well to seal with the surrounding walls, yielding tight coupling. The full-resolution colour activity map in the upper row of Fig. 1*D* allows us to distinguish different retinal areas based solely on the spatial distribution of the activity (e.g. the optic disc in the middle, characterized by its circular structure and lack of spiking activity). The different ‘lobes’ of the activity reflect different parts of the retina, separated by incisions made to flatten the tissue on the array. Overall, the activity propagates laterally from left to right (with a small additional initiation point on the right side after 1–2 s), and then towards both the top and bottom before it evanesces. The bottom row of Fig. 1*D* represents exactly the same episode after downsampling the resolution to an 8 × 8 array, as used in most previous studies. Here, the fine spatial information is lost and it is difficult to accurately follow the path of the activity. (Movies S2, S3 and S6 also illustrate the downsampling effect.)

We used several metrics to characterize the spatiotemporal properties of the activity (see Methods for details), as illustrated in Fig. 2*A*–*E* for a 60 min dataset from a P4 retina. Figure 2*A* shows the raster plot of all detected spikes recorded during 10 min. Following burst detection (Hennig *et al*. 2009), waves are found by grouping spatially contingent channels bursting in synchrony. Figure 2*B* shows the raster plot of detected bursts and waves (each wave in a different colour) for the same time range as in Fig. 2*A*. Figure 2*C* shows the projection plots of two waves (waves A and B in Fig. 2*B*) over the surface of the array. As in Fig. 1*D*, the optic disc area is easily identifiable. (Movie S2 shows a video clip of waves in that retina.)

**Figure 2:**
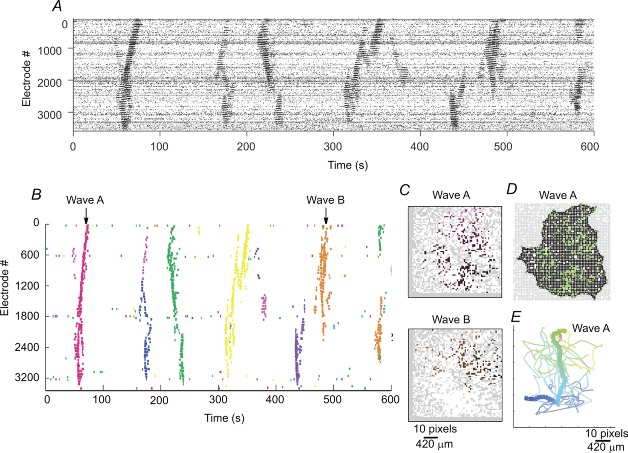
*A*, raster plot of all detected spikes during 10 min recording in a P4 retina. *B*, detected bursts and waves (individual waves illustrated in different colours) in the same time range. *C*, two-dimensional projection of two waves (waves A and B in *B*) over the surface of the chip. Dark colours show channels becoming active first; light colours show channels becoming active last. White and grey dots, respectively, represent all channels firing above and below 0.01 Hz during the recording session. *D*, spatial extent of wave A, estimated using alpha hull analysis. Green dots represent bursting channels participating in the wave; black dots represent channels that do not participate; grey dots outside the wave perimeter represent channels firing at ≥0.01 Hz; white dots represent channels firing at ≤0.01 Hz. *E*, centre of activity trajectories (CATs) of 11 waves belonging to the same cluster as wave A (thick line). The dark blue extremity of the CAT represents the wave initiation site; the warmest colour of the CAT represents wave termination.

One metric used to calculate the wave spatial extent is area, based on alpha hull analysis. Figure 2*D* shows the area of wave A. Within the wave perimeter (delimited by the solid black line), the green and black dots, respectively, represent bursting and non-bursting channels during the wave. The cellular recruitment during a wave is the total number of bursting channels divided by the wave area (activity density). For wave A, the activity density is low, indicating that many RGCs are not recruited as the activity propagates across the retina.

A further measure of wave size is provided by the CAT (Gandolfo *et al*., 2010) used to calculate wave lengths and speeds. The CAT of wave A is indicated by a thick line in Fig. 2*E*.

Finally, to evaluate the randomness of the propagation patterns, we classified all CATs into groups using cluster analysis (Gandolfo *et al*., 2010). CATs with similar propagation patterns are classified as part of the same cluster. Datasets yielding many clusters with few CATs per cluster reflect networks with a high level of randomness in propagation patterns, whereas those yielding few clusters with many CATs per cluster reflect more organized networks with more repetitive propagation patterns. Wave A belongs to a cluster of 11 CATs (Fig. 2*E*).

### Developmental profile of neural activity

We quantified the bursting properties and the spatiotemporal patterns of spontaneous activity during P2–13 (Fig. 3). Interburst intervals (IBIs), burst durations (BDs) and burst sizes (BSs) all show similar developmental profiles, suggesting that important changes underlying firing behaviour occur during P5–8. Indeed, there are fewer but longer bursts during P5–7. They become more frequent and shorter at P8, with a moderate decrease in the number of spikes within bursts. The firing rate within bursts gradually increases during P2–13, with the most significant changes occurring during P10–13.

**Figure 3:**
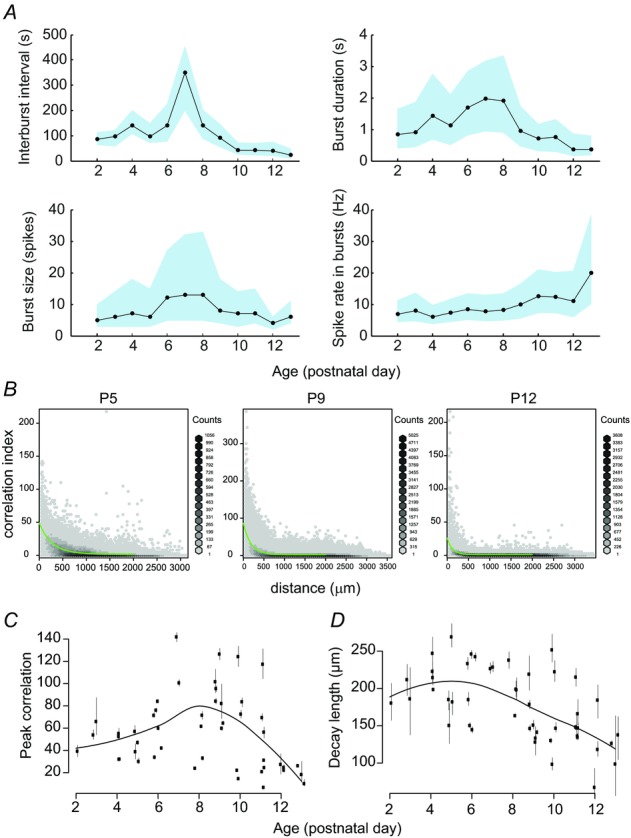
*A*, the median interburst interval (IBI), burst duration (BD), burst size (BS) and firing frequency within bursts as a function of age, pooled for all datasets at each age. Shaded areas denote interquartile ranges. *B*, the correlation index as a function of distance on the retina for single recordings at three ages. Given the large number of cell pairs, we show the density of points by counting the number of points within non-overlapping hexagons of equal size. Darker colours indicate higher counts. The green line shows the fit of the decaying exponential. *C*, *D*, the two key parameters of the fit demonstrated in *B* (peak correlation; decay length) are shown for each recording as a function of postnatal age. The thin vertical lines are the 95% confidence intervals of the parameters. The thick lines are non-parametric locally weighted scatterplot smoothed versions of the data.

To quantify the level of activity synchronization between pairs of electrodes, we calculated the CI (Wong *et al*. 1993) (Fig. 3*B*–*D*). Figure 3*B* illustrates examples from a retina at P5, P9 and P12. One conspicuous difference among these examples is that the maximal CI value virtually doubles during P5–9 and then decreases to a minimum at P12. The second difference is that CI values decay to their minimal values over increasingly shorter inter-electrode distances as development progresses. Peak CI values increase up to P8–9 and then rapidly decrease to their minimum at Stage III (Fig. 3*C*), whereas the decay length of the CI profile increases up to P6–7 and then gradually decreases as the retina matures (Fig. 3*D*). In other words, the activity is highly synchronized over long distances in early Stage II. At the same time, the level of correlation between immediate neighbours increases with development and reaches a peak at P9, which is followed by a significant decrease in synchrony over short distances as development progresses. Figure S1 (online) also provides CI plots for each developmental age.

An advantage of extracellular recordings is that each electrode can record from multiple units. However, in high-density devices such as the APS, the close proximity of neighbouring electrodes (electrode pitch of 42 *μ*m) may result in recording from the same unit simultaneously on several neighbouring channels. To address this issue, for every electrode we calculated the incidence of coincident spikes (e.g. spikes with exactly the same time stamp) between that electrode and its four nearest neighbours. The results of this analysis are shown in Fig. 4, which illustrates the percentage of electrodes for which fewer than 5% (red symbols), 10% (green symbols) and 25% (blue symbols) of the total number of spikes occur in coincidence in at least one of the neighbouring channels. There is a marked increase in coincident spikes with development, demonstrating that the low CI values in young retinas and peak CI values around P8 are not artefacts attributable to the high-density configuration of the array, but, rather, reflect changes in network connectivity with development. Moreover, if spike coincidence purely reflected the proximity of neighbouring electrodes, we would not expect to see developmental changes in activity correlations.

**Figure 4:**
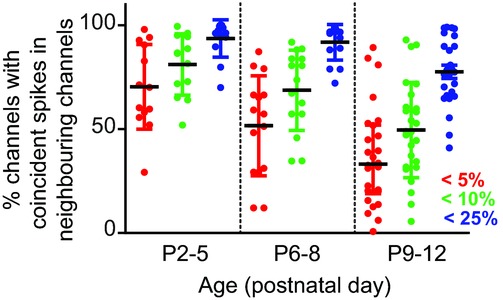
The plot illustrates the percentage of electrodes for which coincident spikes were detected on at least one of their four nearest neighbours for all retinas at all developmental stages during postnatal days (P) 2–12 grouped for P2–5, P6–8 and P9–12. Red, green and blue circles, respectively, indicate the percentage of electrodes with fewer than 5%, 10% and 25% of the total number of spikes occurring in coincidence with neighbours. Individual values represent the mean percentage of channels for a given retina; the horizontal black line represents the mean of all retinas in each age group. Error bars: s.e.m.

### Spatiotemporal wave properties

Figure 5 summarizes the spatiotemporal properties of activity during P2–15. Movies of waves at P4 (Movie S2), P6 (Movie S3), P9 (Movie S4), P10 (Movie S6) and P11 (Movie S7) are available as part of the supporting information (online). Examples of detected waves at Stage II (P5), just before the onset of Stage III (P9) and at the time of eye opening (P12) reveal profound changes in activity patterns (Fig. 5*A*). At P5, both small and large waves invade substantial retinal areas (see also P4 waves in Fig. 2). A striking feature of these early waves is the low density of activity (i.e. relatively few cells are recruited along each wave). Waves gradually become more frequent with development; they shrink and become much denser, and most neighbouring cells are recruited within each wave. At P12, waves become even smaller and then disappear immediately after eye opening; we never recorded activity that could be classified as representing a wave beyond eye opening. Many RGCs burst intensely, but global activity does not follow an organized pattern (Fig. 5*F*).

**Figure 5:**
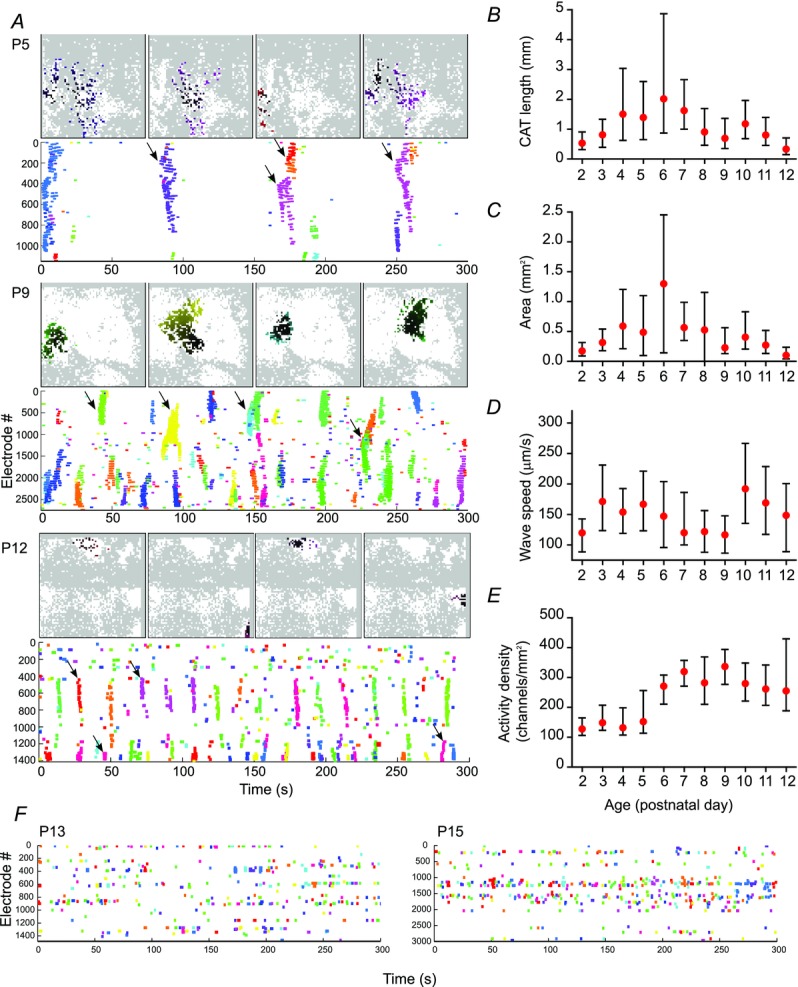
*A*, examples of waves at postnatal day (P) 5, P9 and P12. Each plot shows a raster of detected bursts, with waves colour-coded as in Fig. 1, and two-dimensional projections of selected waves, as indicated by arrows. *B*–*E*, median wave centre of activity trajectory (CAT) length (*B*), area (*C*), propagation speed (*D*) and activity density (*E*) for all waves at all developmental stages [postnatal day (P) 2: one retina, 106 waves; P3: two retinas, 191 waves; P4: four retinas, 204 waves; P5: four retinas, 188 waves; P6: five retinas, 99 waves; P7: three retinas, 72 waves; P8: four retinas, 232 waves; P9: eight retinas, 659 waves; P10: seven retinas, 631 waves; P11: six retinas, 945 waves; P12: four retinas, 718 waves]. Error bars indicate interquartile ranges. *F*, rasters of burst activity recorded at P13 and P15. No propagating activity can be seen at these ages.

Figure 5*B*–*E* summarizes developmental changes in wave CAT length, area, propagation speed and activity density. All four parameters show significant changes (*P* < 0.0001, Kruskal–Wallis one-way ANOVA). Both CAT lengths and areas show similar developmental profiles, indicating that these two metrics are equally effective in measuring wave sizes. Waves grow until P6 and then shrink significantly during P6–9. Once they switch to Stage III, waves become significantly larger at P10 (*P* < 0.05, Dunn's multiple comparison test), and then gradually shrink again until they disappear after P12, reiterating the changes in correlation length illustrated in Fig. 3*D*.

Wave speeds peak at P3 and significantly slow down at P6, becoming slowest during P7–9. However, Stage III waves become faster again, reaching the highest speeds recorded throughout ontogeny. Activity density is low until P5, and then quickly increases at P6–7, remaining high until P12, although it reaches peak values at P9 and undergoes a small but significant decrease in Stage III. The developmental profile of the activity density reinforces the unlikelihood that activity synchronization between neighbours reflects the high density of the electrodes. Indeed, activity density is low until P5, demonstrating that few neighbouring channels are recruited within these early waves.

We also tested whether early cholinergic waves show a directional bias as reported by Stafford *et al*. (2009). To this end, we collected all CAT segments from each single recording session in a circular histogram and used Kuiper's test to establish deviations from circularity in this distribution. In all cases, there was a clear bias of propagation in one orientation, which was highly significant (*P* < 3 * 10^−5^; 12 retinas aged P4–6; data not illustrated). As we did not record the orientation of retinas on the MEA, we were unable to confirm whether this bias did indeed refer to the nasotemporal axis as reported by Stafford *et al*. (2009).

### GABA modulates waves throughout Stages II and III

Our results so far indicate significant changes in excitability and activity patterns during P6–8, coinciding with the shift in the polarity of GABA_A_ signalling in RGCs. We investigated the effect of the GABA_A_ antagonist bicuculline on wave dynamics at different developmental stages. Figure 6*A* shows how P9 waves expand in the presence of bicuculline (10 *μ*m). Blocking GABA_A_ synthesis with the glutamic acid decarboxylase inhibitor l-allylglycine has similar effects (see Movie S5; see also Chabrol *et al*. 2012). Figure 6*B* summarizes the effects of bicuculline on wave parameters at P6 (before the polarity switch), P9 (after the completion of the polarity switch) and at P12 (just before wave disappearance), demonstrating a significant increase in wave size at all three ages (Hennig *et al*. 2011), with maximal effect at P9 (Mann–Whitney one-tailed test). Wave speeds increase at P6 and P9, but not at P12. These results demonstrate that developing GABAergic connections and the polarity of GABA_A_ responses influence wave dynamics, and their effects are maximal once GABA shifts to its mature hyperpolarizing/inhibitory role.

**Figure 6:**
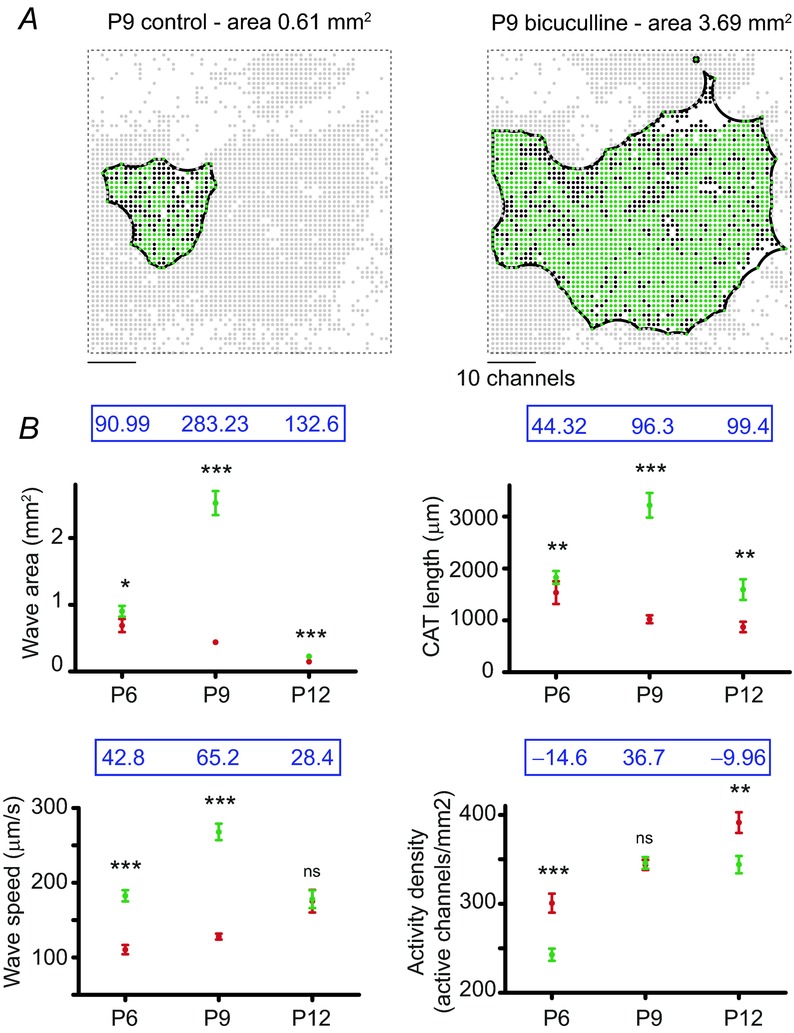
*A*, examples of a wave at postnatal day (P) 9 under control conditions and in the presence of bicuculline (10 *μ*m). Colour coding is as in Fig. 2*D*. *B*, summary of the effects of bicuculline on wave parameters (centre of activity trajectory length, area, speed and activity density) at P6, P9 and P12. Red symbols (mean ± s.e.m.) represent control values; green symbols represent values measured in the presence of bicuculline (P6: 42 and 102 waves for control and bicuculline, respectively; P9: 173 and 94; P12: 272 and 211). Blue numbers above each graph represent the average percentage change for each respective parameter for experiments pooled for P4–6 above P6 (five retinas), P9 (four retinas) above P9, and P10–12 (six retinas) above P12.

### Stage III waves exhibit non-random propagation patterns

We performed cluster analysis of CATs to investigate the level of randomness in propagation patterns (Fig. 7). Waves become less random with development, as shown in Fig. 7*A* for P4 (same retina as in Fig. 2), P10 and P11 retinas. All P4 waves clustered into 10 classes with distinct shapes and start/end points, but overlapped across a substantial fraction of the retina (blue overall activity maps in Fig. 7*A*, right). The number of clusters dramatically decreases with development, coinciding with the substantial drop in spatial extent observed at Stage III, as illustrated in the P10 (three clusters) and P11 (five clusters) examples (Fig. 7*A*). These fewer clusters are also much more segregated spatially; they do not overlap and approximately match the layout of their respective overall activity map. In other words, the activity becomes much less random; it consists of few repetitive patterns that appear to tile the retina rather than overlapping over large retinal areas as at Stage II. These activity ‘hotspots’ do not collectively tile the retina, an issue we address in Figs 8 and 9. Except for a small dip around P6–7, the number of clusters gradually decreases with development (Fig. 7*B*), reaching a minimum at Stage III. In tandem, the fraction of the total number of waves/class increases with development (Fig. 7*C*). Figure 7*D* illustrates the developmental changes in the number of classes and the fraction of waves/class on the same plot, emphasizing their dependency.

**Figure 7:**
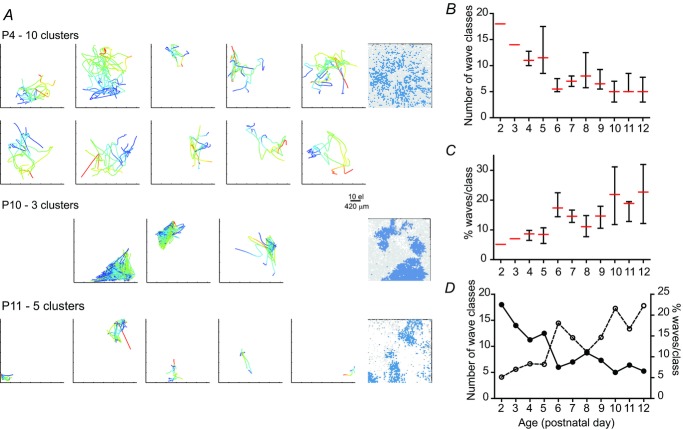
*A*, examples of centre of activity trajectory (CAT) cluster analysis for postnatal day (P) 4, P10 and P11 retinas. Each plot shows all CATs assigned to the same cluster, with colour coding as in Fig. 2*E*. *B*, *C*, the median number of clusters detected (*B*) and fraction of waves per cluster (*C*) as a function of developmental age. Error bars show interquartile ranges. The reduction in cluster number and increase in relative cluster occupancy show that waves become more repetitive and localized with development. *D*, data from *B* and *C* in the same plot, illustrating their dependency.

### Activity hotspots are non-stationary during Stage III

The stereotypical localized activity patterns observed at Stage III caused us to consider if these hotspots identified by clustering are static over time, which would provide strongly biased activity patterns to the brain or retinal postsynaptic partners, or whether they move across the retina over time. To address this question, long-duration stable *in vitro* recordings were performed (as in Hennig *et al*. 2011). We recorded activity in the same retina over 2–3 days in the presence of glutamine (0.5 mm), a precursor of glutamate, to ensure stable glutamatergic activity (Ames & Nesbett, 1981; Brigmann & Euler, 2011). Spontaneous activity was recorded for 30 min approximately every 3 h (except overnight). To control for the health of the retina during these prolonged recordings, we monitored the overall RGC firing rate and measured RGC burst parameters throughout the experiment. Figure 8 illustrates changes in burst duration (circles), burst size (triangles) and overall firing rate (asterisks) for two prolonged recordings, one at P4 (2 days) and one at P11 (3 days). Following an initial increase in all three parameters on day 1 (difference between D1_1 and D1_2), no clear trend was observed in firing patterns over time. There were more fluctuations at P11 than at P4, which is not surprising because at P4 most bursts occur during distinct waves separated by prolonged periods of inactivity (Fig. 10*A*), whereas at P11 the activity consists of small hotspots occurring continuously across the retina (Fig. 9*A*). These results demonstrate that even after 2 or 3 days *in vitro*, RGCs were still capable of generating vigorous bursts. Similar findings were reported in a study looking at wave properties over 2 days during P5–9 (Hennig *et al*. 2011). Figure 9 shows how hotspots move across the retina over 3 days in a retina isolated at P11. The first dataset on day 1 was recorded 4 h after the retina was placed on the MEA (Fig. 9*A*). Different aspects of the synchronization of activity between neighbouring channels are illustrated in three related plots (Fig. 9*B*). The first plot shows the rate of detected putative spikes. We deliberately used a very low detection threshold, which may yield false positives, in order to avoid the mistaken interpretation of fluctuations in tissue coupling over time as moving hotspots (see Methods). The second plot shows the locations of activity hotspots by indicating the probability that the detected spikes occur randomly (independently of the activity in 20 surrounding channels). There are several clear foci of strong synchronized activity and some spikes do not participate in synchronized events (especially in the lower part of the array). The third plot shows the correlation gradient, which indicates where waves or hotspots tend to stop, and the firing similarity index, which indicates the amount of channels exhibiting a similar firing pattern.

**Figure 8:**
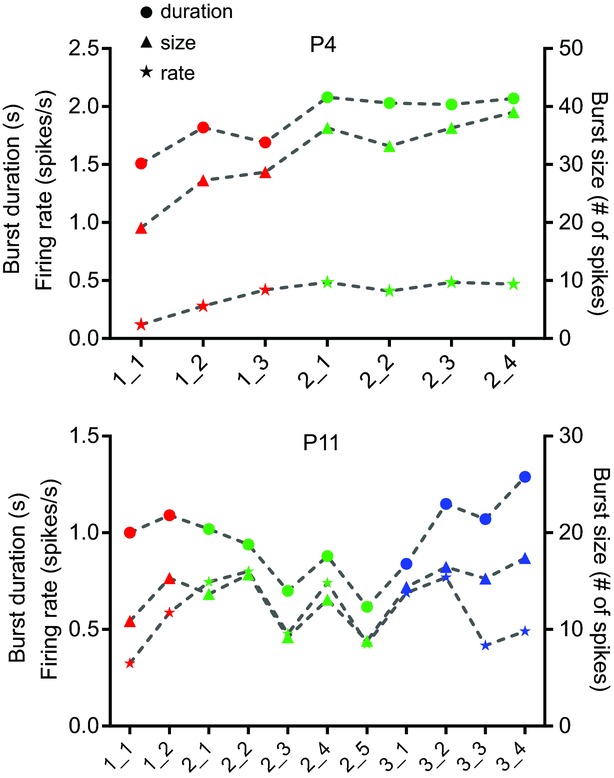
The plots illustrate mean burst durations (circles), burst sizes (triangles) and firing rates calculated across all channels (asterisks) over 2 or 3 days of continuous recording in postnatal day (P) 4 and P11 retinas. Numbers on the *x*-axis represent the day#_recording# (e.g. 1_1 corresponds to Day1_Recording1). Each recording session lasted 30 min and recordings were taken approximately every 3 h, except overnight. The red, green and blue colours, respectively, represent days 1, 2 and 3.

**Figure 9:**
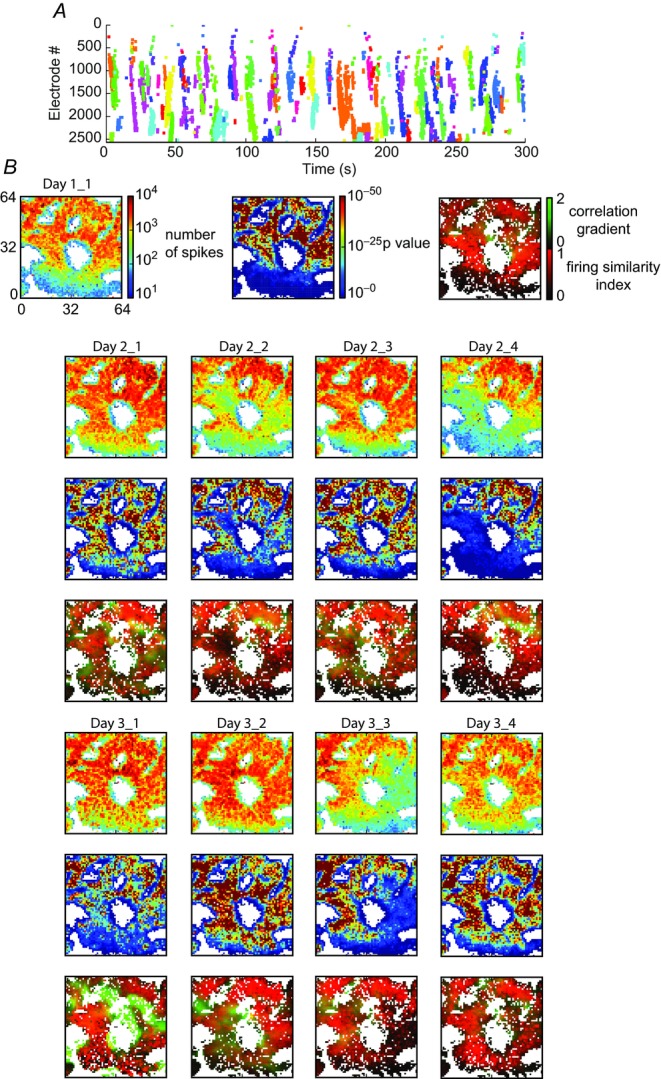
*A*, raster of detected waves from the initial recording trial in a postnatal day (P) 11 retina. *B*, three plots (in vertical order, except for the first dataset in which they are horizontal) show different aspects of the activity for each recording session. The top plot illustrates the raw spike count. The middle plot shows the probability of rejecting the null hypothesis that the activity measured in a channel and its proximal channels are unrelated, thus showing which channels participate in retinal waves and (indirectly via the significance level) how active they are. The darkest red colours represent the lowest probability that the activity is random. The bottom row shows the firing similarity index (red) and the correlation gradient (green) where correlations drop sharply and thus retinal waves tend to terminate.

**Figure 10:**
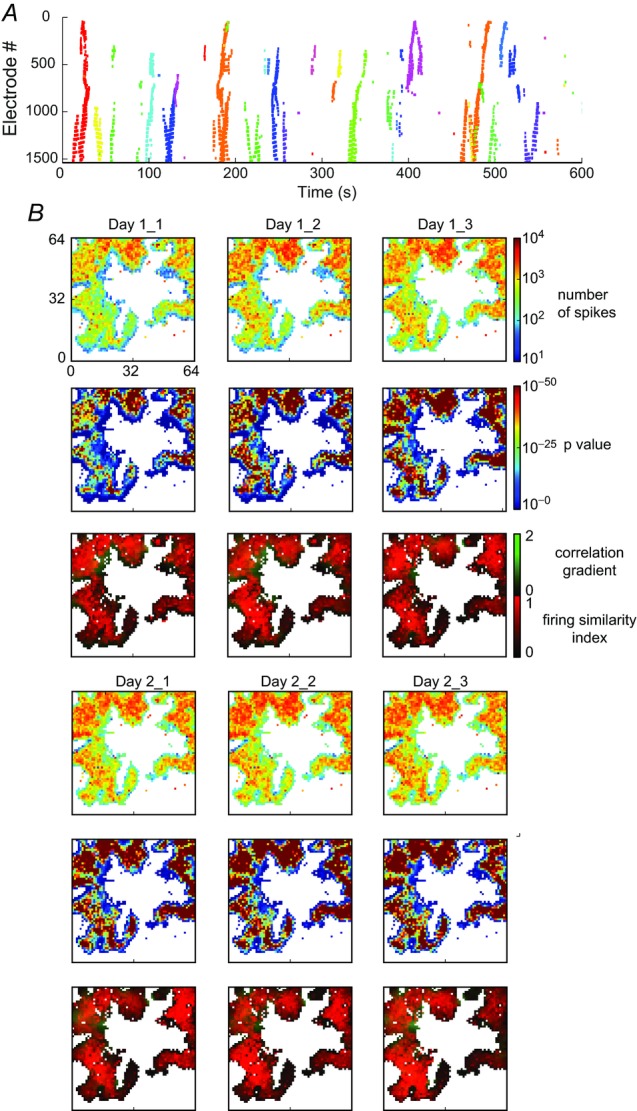
*A*, raster of detected waves in a postnatal day (P) 4 retina. *B*, same analysis as in Fig. 8*B*. Firing activities in different regions do not change differentially over time, and there are no distinct wave boundaries.

Recordings performed over 3 days reveal clear differences in the precise locations of hotspots over time. Importantly, hotspots can reappear in the same location after they disappear (e.g. in the bottom left quadrant of the array), which suggests that their disappearance is not a sign of network dismantlement caused by tissue deterioration. Similar findings were obtained in another P11 and in a P10 retina.

The same approach was applied to Stage II waves, as illustrated in Fig. 10 for a P4 retina. Here, the activity appears much more homogeneous across the retina and no striking differences are observed over time, demonstrating that waves propagate across the entire retinal network over the recording period as previously reported with smaller MEAs (Hennig *et al*. 2011). Similar findings were obtained in a P6 retina.

## Discussion

In this study, we revisited the ontogeny of mammalian retinal waves using the high-density APS MEA (Berdondini *et al*. 2009). Both the overall large size of the array and the spatiotemporal resolution of the recordings reveal novel aspects of the activity. Indeed, our study shows for the first time that: (i) cellular recruitment within waves increases dramatically with development; (ii) waves slow down and shrink when inhibitory GABA becomes involved in their modulation; (iii) glutamatergic waves are faster and much more restricted spatially; (iv) glutamatergic waves are not random like cholinergic waves, and (v) glutamatergic waves occur only in discrete hotspots over the retinal surface and these hotspots move to new locations with time.

In summary, the following developmental picture emerges from our analysis. Shortly after birth, Stage II waves rapidly increase in size and speed. GABA becomes involved in their modulation during P4–5, and the polarity switch beyond P6 coincides with a decrease in wave size and speed and an increase in activity density. Stage III waves emerge at P10; they are very fast, repetitive and focused on activity hotspots that no longer cover the entire retina, but progressively tile the RGC layer. Waves shrink after P10, reaching their lowest sizes at P12, at the time of eye opening, and then disappear.

### High-density MEA recordings

The neonatal mouse eye grows by approximately 30% (from 3.2 mm to 4.3 mm in circumference) during P4–P12 (Wisard *et al*. 2011). Hence, to enable pan-retinal recordings, the active area of the electrode array should cover at least 3.5 mm^2^ at P4 and 5.6 mm^2^ at P12. Most studies currently use 60 channels covering 2.56 mm^2^, corresponding to retinal coverage of 73.1% at P4 and 45.7% at P12. Once programmed cell death is over in the RGC layer (85% complete by P6, 100% complete by P12; Young, 1984; Erkman *et al*. 2000), the mouse retina contains approximately 45,000 RGCs (Jeon *et al*. 1998). Hence, for a P12 retina, a standard 60-channel array will cover 20,565 cells, or 343 cells per electrode. Assuming that each electrode records from four RGCs, this corresponds to a 1.17% yield over roughly 46% of the whole retinal area. Stafford *et al*. (2009) recorded retinal waves using a larger and higher-density array comprising 512 electrodes, but covering only 1.7 mm^2^. Other groups

have recorded from the RGC layer using large, high-density MEA systems (Zeck *et al*. 2011; Fiscella *et al*. 2012). One system uses a 16,384-channel, multi-transistor array covering an area of 1 mm^2^, but signals can be recorded continuously only for 600 ms (Lambacher *et al*. 2011; Zeck *et al*. 2011). The other system contains 11,011 electrodes covering an area of 3.5 mm^2^ (Fiscella *et al*. 2012). However, only 126 channels can be used simultaneously, making it difficult to investigate network events at the pan-retinal level.

In Ca^2+^ imaging studies, the field of view is usually no larger than 0.25 mm^2^ when using sufficiently high magnification to visualize individual RGCs (Sernagor *et al*. 2000, 2003). With the APS MEA, we record from one or two RGCs per electrode during waves (we performed spike sorting for two complete datasets, at P8 and P11, respectively yielding one RGC in 84.3% and 71.0% of the channels, two RGCs in 11.2% and 16.0% of the channels and more than two RGCs in 1.7% and 3.0% of the channels). Hence this system enables us to record simultaneously from ∼10% of the whole mature RGC population spanning the entire retina (the active electrode area measures 7.13 mm^2^) for prolonged periods. The coincidence analysis illustrated in Fig. 4 indicates that the likelihood of recording from the same cell on neighbouring electrodes is low in the APS system; hence our estimation of recording from about ∼10% of the entire RGC population appears reasonable. We attribute the small number of RGCs recorded on each electrode, the low incidence of recording from the same cell on neighbouring electrodes and the high signal-to-noise ratio to the unique design of the electrodes (Fig. 1*A*, inset). The retinal patch coupled to the conductive central recessed area of an electrode becomes electrically insulated from neighbouring retinal areas, increasing the size of the signals and reducing the probability of crosstalk between neighbouring electrodes despite their high-density configuration. In support of this, we record smaller spikes and much larger local field potentials when using conventional 60-channel MEAs with planar electrodes.

In summary, the APS MEA provides a substantial improvement over all previous approaches to population recording and reveals important novel aspects of wave dynamics.

### Developmental changes in Stage II wave dynamics

Waves generated during the prolonged cholinergic phase are far from uniform in terms of their spatiotemporal properties. Following a gradual increase in wave size and peak CI from P2 to P6, striking changes in wave dynamics, indicative of the development of an inhibitory drive in the network, occur during P6–8. Bursts become less frequent and more prolonged, waves progressively shrink and the decay length of the CI decreases. Wave speeds also gradually decrease from P5 to P7. The stronger effect of the GABA_A_ antagonist bicuculline at P6–9 than at earlier stages indicates that both wave shrinkage and the decrease in propagation speed are attributable to the development of GABAergic inhibition. GABA_A_ activity becomes involved in modulating waves in mouse around P4–5 (Wang *et al*. 2007; Hennig *et al*. 2011). As in other parts of the developing CNS (Ben-Ari *et al*. 2007), GABA_A_ responses are initially depolarizing and shift to become hyperpolarizing between P7 and P9 (Zhang *et al*. 2006; Barkis *et al*. 2010), precisely when we see the major changes in wave dynamics. Therefore, it is reasonable to assume that these changes do indeed reflect the maturation of GABAergic inhibition. A similar phenomenon has been demonstrated in turtle retina (Sernagor *et al*. 2003), in which waves gradually slow down and shrink to small activity patches when GABA_A_ signalling becomes functional in the network and subsequently shifts polarity. When the GABA polarity switch is prevented, waves persist (Sernagor *et al*. 2003; Leitch *et al*. 2005), and when endogenous retinal GABA stores are depleted, the small activity patches revert to large propagating waves (see Movie S5) (Chabrol *et al*. 2012). In mammals, GABA_A_ responses also switch polarity around the time waves stop propagating (rabbit: Zheng *et al*. 2004; see also Zhou, 2001; Syed *et al*. 2004b; ferret: Fischer *et al*. 1998).

Stage II waves do not recruit all RGCs on their trajectories, but recruitment increases at P6–7 and remains high until waves disappear. At the same time, the CI gradually increases and peaks around P8–9. This increase in local cellular recruitment within waves is unlikely to reflect the strengthening of cholinergic connections in the inner plexiform layer because there is a gradual increase in the strength of cholinergic immunolabelling during the first postnatal month (Stacy & Wong, 2002; Zhang *et al*. 2005), without any striking changes at P6–7 [although a subtype of RGCs becomes more closely fasciculated with the dendritic processes of starburst amacrine cells by P7 (Stacy & Wong, 2002)]. A possible explanation for this increase at P6–7 is that apoptotic cells do not fire and/or do not participate in waves. In mouse, about 85% of RGC programmed cell death reaches completion at that age (Young, 1984), and residual, slower apoptosis occurs during the second postnatal week (Erkman *et al*. 2000). Moreover, the rate of cell death is much faster during P0–7 than it is later on. Although we know of no experimental evidence of a difference in electrical activity and firing behaviour between dying and surviving RGCs, those RGCs that are destined to undergo apoptosis may not participate in waves.

### Stage III wave dynamics

A recent study of spontaneous activity at Stage III has shown that waves propagate through direct glutamatergic connections and gap junctions between neighbouring ON bipolar cells (Akrough & Kirschensteiner, 2013). This finding may explain why, in our experiments, waves reached their fastest propagation speeds at Stage III. Surprisingly, despite being faster, Stage III waves did not differ much from late Stage II waves in terms of size and activity density. However, the cluster analysis indicates that they were significantly less random than Stage II waves. A closer look at activity patterns recorded within a typical 30 min session reveals few, highly repetitive and non-overlapping patterns, and additional recordings from the same retina over a prolonged period (2–3 days) indicate that the locations of these hotspots were not static. When the activity maps obtained in successive recordings over several days are combined, the overall activity appears to tile the entire retina like a mosaic. Although we have no proof that such movement of hotspots reflects what happens *in vivo* while the retina develops, we do not see the same behaviour at Stage II, in which activity is more or less uniform across the retinal surface over 2 days of recording and does not change with time (Fig. 10) (Hennig *et al*. 2011).

### Developmental relevance of changing wave dynamics

The findings of this study lead us to propose new ideas about how retinal waves may influence the wiring of connections throughout the mouse visual system during development. Eye-specific segregation in the dorsolateral geniculate nucleus (dLGN) and the refinement of topographic maps both occur before the disappearance of Stage II waves and are broadly established by P7 (Muir-Robinson *et al*. 2002; Jaubert-Miazza *et al*. 2005). The present study clearly demonstrates changing dynamics in Stage II waves over time; hence different aspects of this early cholinergic activity may drive various aspects of the innervation of retinal targets. Eye-specific segregation requires competition between both eyes. Hence, it is important for each wave to recruit most of the retina, ensuring synchronization between inputs originating from the same eye. The large and relatively infrequent waves present before P7 provide an excellent substrate through which to achieve this goal. Studies which found wave size to be altered in transgenic mice (Xu *et al*. 2011) support this notion that large waves are required to drive eye-specific segregation.

The refinement of visual maps, however, requires highly correlated activity between immediate neighbours, but not throughout large retinal areas. The smaller and denser waves with highly correlated activity recorded from P7 provide the right conditions for this phase of wiring of visual connections. At the same time, the low density of the earlier waves would ensure that visual maps end up being sharp because such diffuse activity can prevent synchronization between immediate neighbours over widespread retinal areas.

Stage III waves occur after the innervation of retinal targets is established, although termination zones continue to refine in the second postnatal week (Muir-Robinson *et al*. 2002; McLaughlin *et al*. 2003; Jaubert-Miazza *et al*. 2005) and synaptic remodelling in the dLGN during P11–16 is dependent on spontaneous activity (Hooks & Chen, 2006). As topographic maps are broadly established at the time of Stage III waves, what might be the role of such stereotyped activity? We propose that these small, repetitive, non-random hotspots may act as precursors of functional receptive fields in RGCs. Indeed, Stage III waves use the same inner retinal circuitry that is used once visual stimuli are processed through the photoreceptors–bipolar cells–RGCs pathway. Hence these small glutamatergic hotspots may drive activity-dependent processes to strengthen the functional connectivity of individual RGC receptive fields. In support of this, we find that these hotspots tile the retina over time, ensuring homogeneous functional coverage. Interestingly, in the *Crx*−/− mouse (Furukawa *et al*. 1997), a model of retinal degeneration leading to blindness from the onset of visual experience, we found that spontaneous activity becomes disrupted precisely when Stage III waves should emerge, at P10 (see examples in Fig. S1, online), and normal Stage III waves never develop. Finally, MEA recordings of light responses in rat RGCs immediately following eye opening show that receptive field mosaics are already present (Anischenko *et al*. 2010), which suggests that a mechanism to strengthen these connections is already at work during Stage III waves, before visual experience is possible. Testing this hypothesis would require specific manipulation of Stage III wave dynamics and subsequent assessment of light-evoked responses.

## Key points

- Novel pan-retinal recordings of mouse retinal waves were obtained at near cellular resolution using a large-scale, high-density array of 4096 electrodes to investigate changes in wave spatiotemporal properties from postnatal day 2 to eye opening.
- Early cholinergic waves are large, slow and random, with low cellular recruitment.
- A developmental shift in GABA_A_ signalling from depolarizing to hyperpolarizing influences the dynamics of cholinergic waves.
- Glutamatergic waves that occur just before eye opening are focused, faster, denser, non-random and repetitive.
- These results provide a new, deeper understanding of developmental changes in retinal spontaneous activity patterns, which will help researchers in the investigation of the role of early retinal activity during wiring of the visual system.

## Abbreviations

APS: active pixel sensor
BD: burst duration
BS: burst size
CA: centre of activity
CAT: centre of activity trajectory
CI: correlation index
dLGN: dorsolateral geniculate nucleus
IBI: interburst interval
ISI: interspike interval
MEA: multielectrode array
P: postnatal day
RGC: retinal ganglion cell
TS: time step
TW: time window.
