# Supplementary material for: Following the ontogeny of retinal waves: pan-retinal recordings of population dynamics in the neonatal mouse
Source: J Physiol. 2014 Jan 31;592(Pt 7):1545–63. doi: 10.1113/jphysiol.2013.262840 (PMC3979611; doi:10.1113/jphysiol.2013.262840)
Supplement: Supplementary file 1 [file tjp0592-1545-sd1.zip › Supplementary movie legends.docx]

**Movie legends**

**MovieSuppl 1:** this movie shows a short sequence of raw spontaneous activity recorded from a P11 retina in the presence of 10 μM bicuculline (same retina as in Fig. 1 in the main paper). The activity is colour-coded according to the minimum-maximum variations in voltage within 10 ms windows, with blue shades for no activity and warm colour showing subthreshold events and spikes. This retina was particularly active, with repetitive waves flowing towards and away from the optic disc (non-active area in the middle of the field). The total episode real time is 33s.

**MovieSuppl 2:** this movie shows waves detected in a P4 retina (same as in Fig. 2 in the main paper). Wave detection is based on a combination of burst temporal overlap and spatial proximity criteria (see Methods for details). The activity is shown in 1 s bins. Each separate wave is in a different colour with decreasing brightness indicating time. The black dots show channels on which bursts are detected at any time during the entire dataset (which is longer than this video clip) and the grey background represents all non-bursting (or non-active at all) channels on the array. The grey perimeter is the limit of the active area of the chip, measuring 2.67 mm x 2.67 mm. The left panel shows the activity detected at full resolution on the APS MEA and the right panel shows exactly the same dataset, but after downsampling the activity to an 8 x 8 array encompassing the same area as the APS MEA. Each electrode in this case covers 2x2 APS electrodes (42 μm x 42 μm) with a center-to-center separation of 240 μm (~ 6 APS channels). There is a mixture of small and large waves at that age, and the activity density is low. After downsampling it is still possible to see overall activity propagation across the RGC layer, but with significantly less accuracy.

**MovieSuppl 3:** this movie shows waves detected in a P6 retina. See legend of MovieSuppl 2 for more details. The striking difference between P4 and P6 is that here, at P6, the activity density is significantly higher, although this important difference is difficult to establish when comparing the low density recordings in the right panels, emphasizing the advantage of the high density system.

**MovieSuppl 4:** this movie shows waves detected in a P9 retina with the high resolution APS MEA. See legend of MovieSuppl 2 for more details. Most waves are relatively spatially restricted on the array.

**MovieSuppl 5:** this movie shows waves detected in the same P9 retina as in MovieSuppl 4, but following overnight incubation in 2 mM L-allylglycine, an inhibitor of glutamic acid decarboxylase, the enzyme responsible for GABA synthesis. L-allylglycine leads to depletion of endogenous GABA (see Chabrol *et al.* 2012). Waves are significantly larger and faster than in control conditions, demonstrating the important role of GABA in dampening wave propagation across the RGC layer.

**MovieSuppl 6:** this movie shows waves detected in a P10 retina (same as in Fig. 7 in the main paper). See legend of MovieSuppl 2 for more details. These Stage III waves are significantly denser and more restricted spatially than early Stage II waves. Moreover, the activity hotspots always occur in the same retinal areas, they do not tile the retina over the space of a few minutes. The downsampling movie once again demonstrates that the key changes in wave dynamics occurring at this stage are hidden when the activity is sampled at low spatial resolution.

**MovieSuppl 7:** this movie shows waves detected in a P11 retina (same as in Fig. 8 in the main paper) with the high resolution APS MEA. The activity consists mostly of small hotspots.
