# Supplementary material for: Following the ontogeny of retinal waves: pan-retinal recordings of population dynamics in the neonatal mouse
Source: J Physiol. 2014 Jan 31;592(Pt 7):1545–63. doi: 10.1113/jphysiol.2013.262840 (PMC3979611; doi:10.1113/jphysiol.2013.262840)
Supplement: Supplementary file 1 [file tjp0592-1545-sd1.zip › Supplementary text aps2013_hdf.pdf]

HDF5 Datasets for Maccione et al. (2013) manuscript

Stephen Eglen

November 9, 2013

1 Introduction

This document shows how to read and use the multielectrode array data collected for the APS project (Maccione et al., 2013). The data are stored in HDF5 format. This open format was chosen because it can be easily read in different languages (including Matlab and R) and can transparently compress data.

2 CARMEN data storage

We have used the CARMEN system, a virtual laboratory for neuroscientists, to share data and computational services. See `https://portal.carmen.org.uk/`. A username and login has been created to allow the journal editor and reviewers to anonymously view the data files, rather than creating their own new account. Please login as username `aps1` and password `RPfmb235`. (We cannot check who will login using this account, or their IP address.) Whilst the paper is under review, access to the data is currently restricted to users via this account. We will allow unrestricted access when the paper is published.

Once you have logged into the CARMEN system, the data are in the folder "Data -> Shared -> Stephen Eglen -> apsh5". This folder contains the key files for the project.

2.1 Meta-data

The file `00apsdata.csv` is a simple spreadsheet with 48 rows and 2 columns. (1) file – name of the file; (2) age — the age (postnatal days) of the mouse at the time of recording. The CARMEN system also contains some minimal meta-data regarding the recordings. (Two data files, `P7_16June11_m1r1_SpkTs_bursts_filtered.h5` and `P10_6April11_control_SpkTs_bursts_filtered.h5` have been uploaded but not analysed due to abnormal rasters.)

3 Summary of APS data files

The following fields are provided in the data file:

1. **epos**: an Nx2 matrix, where N is the number of electrodes in the current recording. Row i stores the (x,y) grid location of electrode i in this recording. The electrodes are spaced 42 microns apart on a square grid.
2. **sCount**: a vector of length N. sCount[i] stores the number of spikes detected on electrode i.
3. **spikes**: a vector of length S, where $S = sum(sCount)$. These are the spike times (in seconds). The spike trains for each electrode are concatenated into one long vector, so that the spikes for electrode $j \in [1, N]$ are stored in elements a to b, where $a = \sum_{i=1}^{j-1} sCount[i]$ and $b = a + sCount[j] - 1$.
4. **validChannels**: a vector of length at most N, and normally smaller. This contains the indexes of the electrodes that are regarded as having bursty firing.

This is verified by looking at an example file with a unix tool:

```
h5ls P03_AllPhases_Spikes_bursts_filtered.h5

## epos                      Dataset {2, 2093}
## sCount                    Dataset {2093, 1}
## spikes                    Dataset {1, 163710}
## validChannels             Dataset {518, 1}
```

4 Using R to read in HDF5 data

4.1 The rhdf5 package

To access HDF5 data in R, I recommend the rhdf5 package. This is freely available from Bioconductor, and can be installed using the following two lines.

```
source("http://bioconductor.org/biocLite.R")
biocLite("rhdf5")
```

This package does not require any external libraries, as all of the HDF5 libraries are bundled as part of this package. For more details see `http://www.bioconductor.org/packages/devel/bioc/html/rhdf5.html`

4.2 Reading the data

First of all, we need to load the package.

```
require(rhdf5)

## Loading required package:  rhdf5
```

Now, we can use the R routines to check the data file and load it all in at once.

```
datafile <- "P03_AllPhases_Spikes_bursts_filtered.h5"
h5ls(datafile)

##   group          name       otype dclass        dim
## 0     /          epos H5I_DATASET  FLOAT   2093 x 2
## 1     /        sCount H5I_DATASET  FLOAT   1 x 2093
## 2     /        spikes H5I_DATASET  FLOAT 163710 x 1
## 3     / validChannels H5I_DATASET  FLOAT    1 x 518

x <- h5read(datafile, name = "/")
names(x)

## [1] "epos"          "sCount"        "spikes"        "validChannels"
```

Rather than work with the long `spikes` vector, we first break it down (using the function `chop()`) into a more manegable list of spike times, with element i of the list containing a vector of spikes for electrode i.

```
chop <- function(v, counts) {
    ## chop(9:1, c(3,2,4))
    stopifnot(sum(counts) == length(v))
    end <- cumsum(counts)
    beg <- c(1, 1 + end[-length(end)])
    begend <- cbind(beg, end)
    apply(begend, 1, function(x) v[x[1]:x[2]])
}
spiketimes <- chop(x$spikes, x$sCount)
```

For example, here are the spikes recorded on electrode 3:

```
spiketimes[[3]]

##  [1]  105.2  190.1  298.2  406.5  469.2  470.0  570.0 1123.9 1241.6 1246.8
## [11] 1278.0 1318.1
```

Estimate the mean firing rate for each electrode.

```
duration <- max(x$spikes) - min(x$spikes)  # likely to underestimate duration
nspikes <- sapply(spiketimes, length)  # number of spikes on each electrode
firingrate <- nspikes/duration
firing.bursty <- firingrate[x$validChannels]
firing.nonbursty <- firingrate[-x$validChannels]
```

Is there a difference in the firing rates of electrodes that were bursty versus those that were not bursty? Figure 1 compares the firing rate of the two populations; there are a small number (5) of outliers with firing rates > 0.5 Hz, but we ignore those here.

```
plot(ecdf(firing.bursty), col = "green", xlab = "firing rate (Hz)", bty = "n",
    las = 1, ylab = "CDF", main = "", xlim = c(0, 0.5))
lines(ecdf(firing.nonbursty), col = "blue")
legend("bottomright", legend = c("bursty", "nonbursty"), lty = 1, col = c("green",
    "blue"))
```

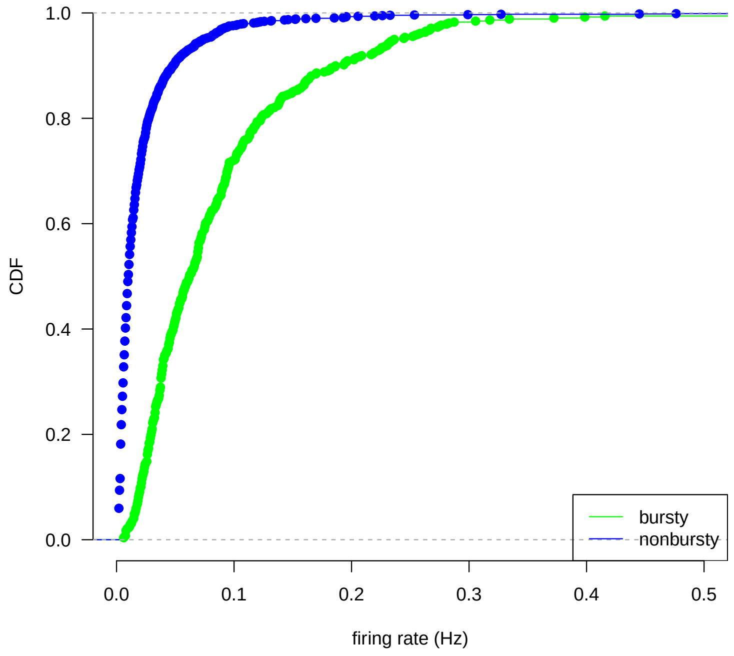

Figure 1: CDF of firing rates of bursty and nonbursrty electrodes (R version).

4.3 Reading a subset of data

One key feature of HDF5 is that you can read in just a section of data, rather than the entire contents at once (useful with very large files). For example, let's retrieve 10 spikes (elements 30 to 39) afresh from the datafile.

```
t(h5read(datafile, "/spikes", index = list(c(30:39), 1)))

##       [,1]  [,2]  [,3]  [,4] [,5]  [,6] [,7]  [,8]  [,9] [,10]
## [1,] 94.79 106.3 110.6 136.3  145 161.4  176 179.8 181.5 237.7
```

5 Using matlab to read in HDF5 data

Below is an example matlab script for reading in the MEA data, in a similar fashion to R. Since about 2010, matlab has had support for reading and writing HDF files. Figure 2 shows the CDF of firing rates for the two populations of neurons.

```
%% Example script to read in the HDF5 data file.
datafile = 'P03_AllPhases_Spikes_bursts_filtered.h5';

%% Check the contents of the file.
h5disp(datafile)
```

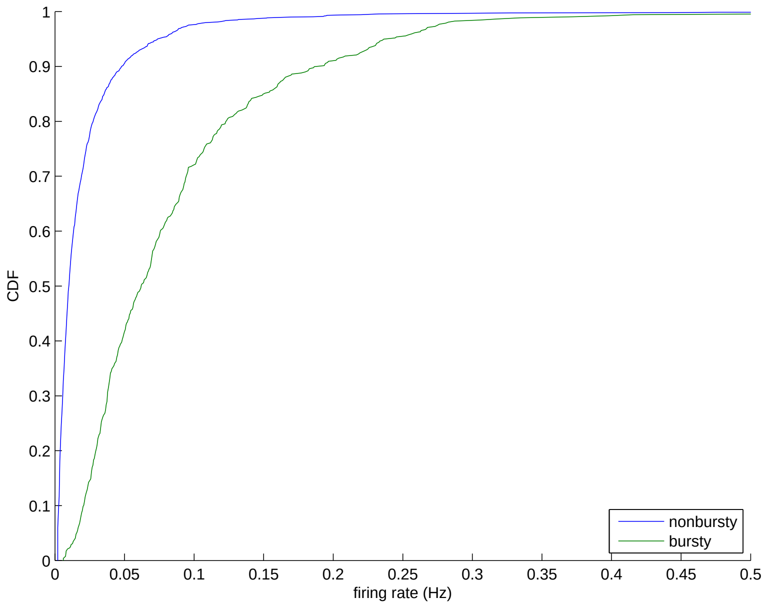

Figure 2: CDF of the firing rates for both bursty and nonbursty electrodes (matlab version).

```
%% Store all the information relating to the file in the struct z.
%% Currently we read the file one item at a time, but hopefully
%% there will be a function to read in everything at once, just like R.
z.epos = h5read(datafile, '/epos');
z.sCount = h5read(datafile, '/sCount');
z.spikes = h5read(datafile, '/spikes');
z.validChannels = h5read(datafile, '/validChannels');


%% Break up the spikes vector into a cellarray, such that spikeimes(i)
%% contains a vector of the spike times for electrode i.
spiketimes = mat2cell(z.spikes, z.sCount, 1);

%% Show the spikes for electrode 3.
spiketimes{3}

%% Guess the duration of the recording
duration = max(z.spikes) - min(z.spikes);
nspikes = cellfun(@length, spiketimes);
firingrate = nspikes / duration;
firingbursty = firingrate(z.validChannels);
N = numel(firingrate);
firingnonbursty = firingrate(setdiff(1:N, z.validChannels));

%% Create a plot with the output.
[f1,x1] = ecdf(firingnonbursty);
[f,x] = ecdf(firingbursty);
p = plot(x1, f1, x, f);
box off
legend(p, 'nonbursty', 'bursty','location', 'southeast');
xlabel('firing rate (Hz)')
xlim([0 0.5])                          %% exclude a few high rate outliers.
ylabel('CDF')
saveas(gcf, 'mymat.pdf', 'pdf')
```

6 Creating HDF5 in matlab

The high-level functions for HDF5 currently require every object to be a numeric object (vectors and matrices are okay, but a cell array, even if it contains only vectors or matrices, is not). This is why the spiketime object is stored as a flat vector. (We could write more code to write one spike train at a time.) The following script reads in matlab .MAT files and creates corresponding HDF5 files.

```
function mat7toh5(matfile, h5file)
    % Convert a matlab file to an h5 file
    load(matfile)

    %% Delete file if it already exists.
    system(sprintf('rm -f %s', h5file));

    h5create(h5file, '/epos', size(epos));
    h5write(h5file, '/epos', epos);

    if (exist('sCount'))
        h5create(h5file, '/sCount', size(sCount));
        h5write(h5file, '/sCount', sCount);
    else
        h5create(h5file, '/sCount', size(scount));
        h5write(h5file, '/sCount', scount);
    end

    if (exist('spiketimes'))
        spikes = vertcat(spiketimes{:});
    else
        spikes = vertcat(spikes.times{:});
    end

    if (not (length(spikes) == sum(sCount)))
        error('spike lengths differ')
    end
    % Compress the spike stream, as it is quite big.
    h5create(h5file, '/spikes', size(spikes), 'Deflate', 9, 'ChunkSize', size(spikes));
    h5write(h5file, '/spikes', spikes);

    h5create(h5file, '/validChannels', size(validChannels));
    h5write(h5file, '/validChannels', validChannels);
end

% Convert a directory of mat files into equivalent HDF files.
% Can convert either mat5 or mat7 files.
matdir = '/tmp/v5matfiltered'
%%matdir = '/local/data/home/stephen/APS'
h5dir = '/tmp/h5'
matfiles = dir([matdir,'/','*mat'])

for i = 1:length(matfiles)
  mat = matfiles(i).name;
  h5name = strrep(mat, '.mat', '.h5')
  m = [matdir, '/', mat]
  h = [h5dir, '/', h5name]
  %%mat2h5(m, h)
  mat7toh5(m, h)
  %%mat7toh5v2(m, h)
end
```

Acknowledgements

Bernd Fischer (R support for HDF5), Johannes Hjorth (comments), Malcolm Lidierth (advice on HDF5).
