# Supplementary material for: Following the ontogeny of retinal waves: pan-retinal recordings of population dynamics in the neonatal mouse
Source: J Physiol. 2014 Jan 31;592(Pt 7):1545–63. doi: 10.1113/jphysiol.2013.262840 (PMC3979611; doi:10.1113/jphysiol.2013.262840)
Supplement: Supplementary file 1 [file tjp0592-1545-sd1.zip › FigSuppl1.docx]

FigSuppl: Correlation index plots for each developmental age. We computed the correlation indexes for each recording. For each developmental age, we then merged the correlation index plots. The number of recordings merged at each age is given in each title. Error bar denotes mean and s.d. for cell pairs at a given distance bin (width = 50 μm). Beyond 1,000 μm, correlation index profiles remained flat.
