# Supplementary material for: Following the ontogeny of retinal waves: pan-retinal recordings of population dynamics in the neonatal mouse
Source: J Physiol. 2014 Jan 31;592(Pt 7):1545–63. doi: 10.1113/jphysiol.2013.262840 (PMC3979611; doi:10.1113/jphysiol.2013.262840)
Supplement: Supplementary file 1 [file tjp0592-1545-sd1.zip › FigSuppl2.docx]

In inherited retinal dystrophic disorders, photoreceptors undergo apoptosis at various postnatal times. Interestingly, remodelling of the neural retina (particularly of glutamatergic connections) is known to start long before photoreceptors begin to die (Marc *et al*. 2003). This motivated us to investigate whether recording spontaneous activity during Stage II and Stage III waves in a model of retinal dystrophy may provide clues about the potential role of Stage III waves in shaping retinal connectivity in an animal that will never experience vision. We have recorded spontaneous activity in retinas isolated from the neonatal cone rod homeobox (Crx) knockout mouse, a model of Leber Congenital Amaurosis (photoreceptors apoptosis occurs between 1-6 months postnatal) (Furukawa *et al*. 1997). The figure below (bottom panel) shows that Stage II waves appear normal in the Crx retina. However, the typical Stage III hotspots observed in the wild type retina (top panel) were completely absent in age-matched Crx-/- retinas. Out of 12 retinas recorded between P10-11, 3 retinas barely expressed correlated spontaneous activity at all, whilst the 9 other retinas showed various levels of activity that lacked any organised patterns (second and third panels). Activity was strong in most cases, with intense and prolonged bursting episodes occurring sometimes over very large retinal areas. The entire RGC layer also develops strong glutamate-mediated ~10Hz oscillations and bursting later on (from ~P15) and lasting throughout the degeneration period (Soto *et al*. 2012), similar to the rd1 mouse (model of *retinitis pigmentosa*; Stasheff, 2008; Menzler & Zeck, 2011).

Furukawa T, Morrow EM & Cepko CL (1997). Crx, a novel otx-like homeobox gene, shows photoreceptor-specific expression and regulates photoreceptor differentiation*. Cell* **91**, 531-541.

Marc RE, Jones BW, Watt CB & Strettoi E (2003). Neural remodeling in retinal degeneration. *Prog Ret Eye Res* **22**, 607-655.

Menzler J & Zeck G (2011). Network oscillations in rod-degenerated mouse retinas. *J Neurosci* **31**, 2280-2291.

Soto F, Ma X, Cecil JL, Vo BQ, Culican SM & Kerschensteiner D (2012). Spontaneous activity promotes synapse formation in a cell-type-dependent manner in the developing retina. *J Neurosci* **32**, 5426-5439.

Stasheff SF (2008). Emergence of sustained spontaneous hyperactivity and temporary preservation of OFF responses in ganglion cells of the retinal degeneration (rd1) mouse. *J Neurophysiol* **99**, 1408-1421.
